# A response to readers’ comments

**DOI:** 10.1186/s12974-018-1300-8

**Published:** 2018-09-14

**Authors:** Zhiyi Zuo, Zhi Wang, Shiyu Meng, Shuling Peng

**Affiliations:** 1 Department of Anesthesiology, Sun-Yat-Sen Memorial Hospital, Sun Yat-Sen University, Guangzhou, 510289 Guangdong China; 2 Laboratory of RNA and Major Diseases of Brain and Heart, Sun Yat-Sen Memorial Hospital, Sun Yat-Sen University, Guangzhou, 510120 Guangzhou China; 3 Department of Anesthesiology, University of Virginia, Charlottesville, VA 22908 USA

**Keywords:** Anesthetic, Brain aging, Inflammasome, Postoperative cognitive dysfunction

## Abstract

This is a response to readers’ comments on our paper entitled “Critical role of NLRP3-caspase-1 pathway in age-dependent isoflurane-induced microglial inflammatory response and cognitive impairment” published in the Journal of Neuroinflammation this year.

To the readers and editor,

We appreciate the comments by Yin et al. The focus of our study was to determine whether the nucleotide binding and oligomerization domain-like receptor family pyrin domain-containing 3 (NLRP3)-caspase 1 pathway played a role in the primed status of brain cells in aging brain and whether this primed status contributed to isoflurane-induced neuroinflammation and cognitive dysfunction. We provided in vitro and in vivo evidence for these effects. To implicate the role of NLRP3-caspase 1 pathway in the isoflurane effects, we used Ac-YVAD-cmk, a caspase 1 inhibitor, and NLRP3 siRNA. We feel confident that caspase 1 activity is important for the production of interleukin (IL)-18 and IL-1β and that NLRP3 activation is critical for isoflurane-induced caspase 1 activation. The evidence to support these points is in figure 2 and figure 6, respectively, of our publication [1].

We agree that MCC950 may be an additional tool to probe the role of NLPR3 in isoflurane effects. The proposal that pyroptosis contributes to isoflurane-induced cognitive impairment is interesting. However, our study did not show any effects on cell viability up to 12 h after NLRP3 was activated. However, it will be interesting to know whether NLRP3 inflammasome-dependent pyroptosis is involved in age-dependent isoflurane-induced cognitive impairment. These interesting ideas/proposals require experiments to test.

## Abbreviations

IL: Interleukin
NLRP3: Nucleotide binding and oligomerization domain-like receptor family pyrin domain-containing 3
